# Cancer Immunotherapy and the Immune Response in Follicular Lymphoma

**DOI:** 10.3389/fonc.2018.00219

**Published:** 2018-06-19

**Authors:** Frank Stenner, Christoph Renner

**Affiliations:** ^1^Department of Oncology, University Hospital Basel, Basel, Switzerland; ^2^Department of Biomedicine, University of Basel, Basel, Switzerland

**Keywords:** follicular lymphoma, indolent lymphoma, monoclonal antibodies, bispecific antibodies, radio-immunotherapy, checkpoint blockade inhibitors, chimeric antigen receptor therapy

## Abstract

Follicular lymphoma (FL) is the most frequent indolent lymphoma in the Western world and is characterized in almost all cases by the t(14;18) translocation that results in overexpression of BCL2, an anti-apoptotic protein. The entity includes a spectrum of subentities that differ from an indolent to a very aggressive growth pattern. As a consequence, treatment can include *watch & wait* up to intensive chemotherapy including allogeneic stem cell transplantation. The immune cell microenvironment has been recognized as a major driver of outcome of FL patients and gene expression profiling has identified a clinically relevant gene expression signature that classifies an immune response to the lymphoma cells. It is known for some time that the immune cell composition of the lymphoma microenvironment is important because high numbers of tissue-infiltrating macrophages correlate with poor outcome in patients receiving chemotherapy but not in patients receiving the combination of chemotherapy and CD20-specific monoclonal antibody rituximab. In addition, TCR signaling of tumor-infiltrating lymphocytes is dysfunctional leading to an impaired capacity to form an intact immunologic synapse. Approaches restoring local T cell function, e.g., by usage of checkpoint inhibitors has demonstrated clinical activity (ORR 40%) and can achieve long-term remissions. Ongoing trials with re-programmed autologous CART cells achieve response rates in approximately 50% of FL patients with relapsed and even refractory disease. Responses lasting for more than 6 months might be durable, indicative for a successful restoration of a functional immune system. In summary, FL is a malignant disease where the control by the immune system ultimately decides about progression and transformation rate. The advent of monoclonal antibodies has changed the way we treat FL and new approaches restoring the individual immune control will hopefully improve results further.

## Introduction

The aim of this review is to present the current therapeutic landscape of follicular lymphoma (FL) and to discuss early results of immunotherapies, e.g., checkpoint inhibitors and CAR T-cell therapies in the context of the immune system.

The clinically established therapeutic options for FL today are mainly focused on cytoreduction. Without the exception of the CD20 targeted therapies the recruitment of the patient’s immune system is not actively utilized in the conventional therapy of FL. However, for long-term benefits, it will be crucial to make progress in that direction, otherwise FL will continue to be an incurable and chronic disease. Thus, understanding the interplay between FL cells and their environment will be key for further success in this disease.

Other than in aggressive B-cell lymphomas like diffuse large B-cell lymphoma (DLBCL), the principle structure of the lymph node is more conserved in FL. In FL, the lymph node architecture is not destroyed, and the nodes boundaries are better respected by the tumor. The longevity of the malign B-cell, more than the aggressive behavior of these cells leads to abnormal large follicles. This phenotype is a result of the increase of lymphocyte numbers in the germinal centers that swell due to the sheer load. In FL, the often slow progression also leads into a long-standing relationship of the increasing B-cell numbers and their neighboring immune cells and the stromal microenvironment. This results inevitably in shifts and alterations within the immune microcosm. T-cells in FL lesions have been found to be increased (Zhang/Ansell); however, these T-cells, when examined in detail, often display features of T-cell exhaustion, for example, high expression of PD-1 or TIM-3 (1). Putative tumor supporting T-cells from T-cell subsets especially T-helper cells, T-cells (Tregs), and there most prominently follicular regulatory T-helper cells (FOXP3+) become involved in the protection of the malignant FL clone and foster its immune evasion.

The importance of an effective T-cell surveillance in the context of lymphoma has been demonstrated in various mouse models. For example, immunodeficient mice that lack T-cell or NK cell effector molecules like perforin or IFN-γ develop spontaneous lymphomas. These lymphomas when transferred on wild-type littermates are immediately rejected by CD8 positive T-cells (2, 3). In summary, suppression of a T-cell-mediated antitumor response appears to be instrumental for the initial establishment and further development of FL.

With a remarkable variability of clinical courses in FL, several efforts to better predict outcome according to biological features of the individual disease have been made. A gene expression-based model has identified two subsets of immune signatures in FL with distinct biologic attributes in FL that are associated with survival (4). These specific signatures were not expressed in the malign or benign B-cells but the genes in the immune response 1 signature were more highly expressed in T cells than in any of the B-cell or monocyte subpopulations, and genes of immune response 2 were more pronounced expressed in both T cells and monocytes but not in B-cells. Patients with immune signature 1 had a better outcome than those of immune signature 2, underscoring an important contribution of monocytes for a more dismal outcome in FL.

The role of monocytes in FL was further substantiated by a study (5) that found upregulated CCR1 and CD68-positive immune cells within FL lesions indicating a monocytes and macrophages recruitment. This pattern was apparently associated with worse survival in FL. In contrary, higher numbers of T-cells with elevated levels of CD3 and the early T-cell antigen CD7 were correlated with better survival in the examined cohort. Finally, CD4 and CD8 subsets were not significantly associated with outcome. Both findings are in line with the observation of two distinct immune profiles published by Dave et al. (4). These results confirm the role of the host immune responses for the outcome in FL and specifically demonstrate that the degree of infiltrating CD68 macrophages and CD7-positive T-cells is prognostically useful, together with identification of CCR1 as a putative novel prognostic indicator and a marker for an immune switch between macrophage and T cell-dominant response. With the advent of immune targeted therapies either against tumor supporting T-cells of lymphoma-associated macrophages, the vision of a chemotherapy free regimen for FL comes closer to reality.

## Biology of FL

Follicular lymphoma is among the most frequently occurring entities of indolent non-Hodgkin’s lymphoma. Generally, FL presents as a slowly growing disease, which can be quite asymptomatic for some time. Once clinical problems are noted it is rather by compression of other structures than invasion or destruction of adjacent structures. If FL is detected in early stages (I and II) radiotherapy has curative potential. However, due to frequent bone marrow involvement (stage IV), many patients are not eligible for this curative option. Ultimately, almost all patients will experience relapse, and a proportion of patients will develop an aggressive disease with high risk of transformation. The annual rate of histological transformation in FL patients is estimated with 3% (6). Although advanced FL is considered incurable, recent advances in the treatment and management of this disease have made a significant impact on progression-free survival (PFS) and patient quality of life. Long treatment-free survival intervals in some patients suggest a possible cure in a subset of these patients, but as of today it is too early to make this claim. Even if FL is still considered incurable, affected patients generally have a long median overall survival (OS) that can reach 10 years or more. The advent of monoclonal antibody therapy in conjunction with new chemotherapeutics and the addition of radionuclides in the recent past have had a significant impact on FL management and have resulted in much better outcomes.

## Clinical Presentation and Course of FL

Most patients initially present with asymptomatic peripheral lymphadenopathy, affecting the cervical, axillary, femoral, and inguinal regions (7). Although lymph nodes are most commonly involved, the disease may also originate at or affect certain extranodal sites. These include the duodenum, skin, thyroid, salivary gland, and the breast (8). Stage IV disease is present in approximately two-third of the cases most often demonstrated by involvement of the bone marrow (9). Clinical features like night sweats and weight loss—typically associated with more aggressive forms of lymphomas such as DLBCL—might be present but are often missing even in higher stages of the disease. The ESMO recommendations appreciate the diversity of the FL subtypes, and the therapeutic options for the individual patients should be taken into consideration when planning the appropriate therapy (10).

## Immunochemotherapy and Radiotherapy for FL

Passive immunotherapy, e.g., monoclonal antibodies against CD20, in combination with a chemotherapy backbone is currently the standard of care for patients with advanced-stage FL in need of treatment (10–13). Some patients with a low burden may be treated with CD20-specific antibodies (such as rituximab) only. However, a proportion of patients do not respond to standard treatment, and the majority will relapse after an initial response, highlighting the need for other more effective and durable therapies. An alternative approach to monoclonal antibodies with or without chemotherapy is the usage of radionuclide labeled anti-CD20 antibodies that are described in more details later in the article.

Radiotherapy has a potential to improve PFS and improves OS for FL patients with early clinical stages (I and II) by approximately 15% (14, 15). The standard radiation dose for FL is 24 Gy and has been shown to be superior to 4 Gy delivered as 2 × 2 (FORT trial) (16). However, long-term remissions in advanced FL patients receiving TBI with 2 Gy × 2 Gy and patients who had aborted the full doses for various reasons have been observed. Therefore, given the exquisite radiosensitivity of FL and the presumable added control by the immune system when applying lower doses of radiation suggest that there is a mechanism of radiotherapy beyond sheer lymphoma cell destruction.

### Identification and Characterization of Potential Target Antigens

Being a more mature B-cell disorder, FL displays the immunophenotype of follicular center B-cells. Pan-B-cell markers (CD19, CD20, CD22, and IgM) are present with a co-expression of CD10. In contrast to reactive B-cells, FL cells express BCL-2. The expression of this anti-apoptotic protein due to t(14;18)(q32;q21) event, that brings the BCL-2 gene under the activity of the Ig heavy chain promoter is regarded to be pathognomonic for the disease. For therapeutic purposes, CD20 followed by CD19, CD22, and CD74 appear to be valid targets for immunotherapy (17, 18). While CD20 is a non-internalizing antigen, the latter three are internalized and they have or will be tested in trials utilizing antibody drug conjugates that rely on internalization (Table 1 includes various contemporary approaches in FL).

**Table 1:** Ongoing trials in follicular lymphoma (FL) with immune interventions on clinical.trials.gov.

Spalte1	Study	Condition	Study drug	NCT-ID
1	Sequential intranodal immunotherapy (SIIT) combined with anti-PD1 (pembrolizumab) in follicular lymphoma	Fl and other NHL	Pembrolizumab	NCT02677155

2	Active specific immunotherapy for follicular lymphomas with tumor-derived immunoglobulin idiotype antigen vaccines	Fl and other NHL	Id-KLH vaccine|GM-CSF	NCT00001512

3	Cellular adoptive immunotherapy in treating patients with relapsed or refractory follicular non-Hodgkin’s lymphoma	Fl and other NHL	Aldesleukin plus rituximab	NCT00182650

4	BI 695500 vs rituxan first line treatment in patients with low tumor burden follicular lymphoma	Fl and other NHL	Rituximab|BI 695500	NCT02417129

5	Monoclonal antibody CT-011 in combination with rituximab in patients with relapsed follicular lymphoma	Fl and other NHL	CT-011|rituximab	NCT00904722

6	Rituximab with or without yttrium Y-90 ibritumomab tiuxetan in treating patients with untreated follicular lymphoma	Follicular lymphoma	Rituximab|radiation: yttrium Y-90 ibritumomab tiuxetan	NCT02320292

7	Vaccine therapy plus interleukin-2 in treating patients with stage III, stage IV, or recurrent follicular lymphoma	Fl and other NHL	Aldesleukin|autologous tumor cell vaccine	NCT00020462

8	Zevalin. First line in follicular lymphoma	Follicular lymphoma	90Yttrium-ibritumomab tiuxetan + rituximab; rituximab	NCT00772655

15	Phase I dose escalation study of IMMU-114 (anti-HLA DR) in relapsed or refractory NHL and CLL	Fl and other NHL	IMMU-114	NCT01728207

16	Agatolimod (anti-toll 9 receptor), rituximab, and yttrium Y 90 ibritumomab tiuxetan	Fl and other NHL	Agatolimod sodium|radiation: indium In-111 ibritumomab tiuxetan	NCT00438880

17	Radiolabeled monoclonal antibody plus rituximab with and without filgrastim and interleukin-11	Fl and other NHL	Rituximab|yttrium Y 90 ibritumomab tiuxetan	NCT00012298

19	Epratuzumab (anti-CD22) in treating patients with non-Hodgkin’s lymphoma	Fl and other NHL	Epratuzumab	NCT00022685

20	Denintuzumab mafodotin (SGN-CD19A) combined with RCHOP or RCHP versus RCHOP alone	Fl and other NHL	Denintuzumab mafodotin|rituximab|chemotherapy	NCT02855359

21	Study evaluating the efficacy and safety of PCAR-019 in CD19 positive relapsed or refractory leukemia and lymphoma	Fl and other NHL	PCAR-019 (anti-CD19 CAR-T cells)	NCT02851589

23	Treatment study of denintuzumab mafodotin (SGN-CD19A) plus RICE versus RICE alone for diffuse large B-cell lymphoma	Fl and other NHL	Denintuzumab mafodotin|rituximab|chemotherapy	NCT02592876

24	Immunotherapy with *ex vivo*-expanded cord blood-derived NK cells combined with rituximab HDCT/ASCT for B-NHL	Fl and other NHL	NK cells|rituximab|chemotherapy|ASCT	NCT03019640

25	Idiotype vaccine for low-grade non-Hodgkin’s lymphoma	Fl and other NHL	FavId (Id-KLH) active immunotherapy	NCT00036426

26	Rituxan plus favid (idiotype vaccine) for low-grade non-Hodgkin’s lymphoma	Fl and other NHL	Id-KLH	NCT00041730

*An overview of trials in FL that are currently examining the role of conventional, e.g., non-cellular interventions (antibodies, vaccines, etc.)*.

### Development of Monoclonal CD20-, CD19-, and CD22-Specific Antibodies

Rituximab has been the first monoclonal antibody entering clinical practice in a variety of lymphomas of the B-cell origin. Thus, it was no surprise that rituximab has found an undisputed place in the treatment of FL. Other than in aggressive lymphomas, strategies using monotherapy of rituximab with and without maintenance have been established successfully (19–21). Consequently, guidelines like ESMO recommend to start rituximab in patients in need of therapy but with low tumor burden and slow progression (10). With the advent of type II monoclonal antibodies, namely, obinutuzumab the landscape of treatment begins to shift. Obinutuzumab is a glycoengineered, afucosylated anti-CD20 antibody with increased antibody-dependent cellular cytotoxicity and increased antitumor activity by FCγRIII compared with rituximab or ofatumamab (22). In a phase III trial (GALLIUM), patients were randomized 1:1 to receive either obinutuzumab and chemotherapy or rituximab and chemotherapy, followed in responding patients by obinutuzumab or rituximab maintenance for up to 2 years. There, an advantage of obinutuzumab regarding PFS compared with rituximab was shown (23). In 2017, the Food and Drug Administration approved obinutuzumab (GAZYVA, Genentech, Inc.) in combination with chemotherapy, followed by obinutuzumab monotherapy in patients achieving at least a partial remission, for the treatment of adult patients with previously untreated stage II bulky, III, or IV FL, respectively.

### Antibody-Based Radio-Immunotherapy

Bexxar (131I-tositumomab) and zevalin (90Y-ibritumomab tiuxetan) have been approved in the US and zevalin also in Europe. Both agents can achieve meaningful responses, as shown by an approximately 75% complete response (CR) rate in patients treated with 131I-tositumomab (24). In some cases, these responses lead to long-lasting remissions. Widespread use of these therapies has been hampered by challenging logistics and the restricted availability outside specialized centers. Thus, among the growing list of therapeutic options for FL, the radioimmunotherapeutics lead a shadowy existence.

### Antibody-Based Immunotoxins (ITs)

SGN-CD19B, a PBD conjugated antibody, has shown its best preclinical responses in FL when compared with other B-cell malignancies (25). ITs have the advantage of increased efficacy by reduced toxicity compared with antibody chemotherapy combinations. Whether long-term control of FL like in some patients treated with rituximab monotherapy seen in SAKK 35/98 trial (26) is achievable with antibody-based ITs also has to be seen in future.

## High Dose Chemotherapy Followed by Autologous or Allogenic Stem Cell Transplantation

Transplant concepts found their place in the pre-rituximab era, when relapses were more frequent and swift than after the introduction of the CD20 antibody. Beside recognition of its curative potential transplantation lost ground in the therapeutic algorithm of FL with the introduction of rituximab. Today, it can be regarded consensus to use high-dose chemotherapy followed by autologous stem cell transplantation (HDCT/ASCT) as a salvage treatment. In patients with refractoriness to first-line treatment and transformed lymphomas, this concept should be applied earlier. Allogenic stem cell transplantation has curative potential but carries a mortality risk for patients with FL (16). The benefit of a total reset of the immune system and a graft versus lymphoma effect are undeniable, but the risk involved for the patient is significant. Therefore, a careful upfront risk benefit evaluation should be done. Allogeneic transplantation should be reserved for patients failing of HDCT/ASCT [for an excellent review on this topic, see Ref. (27)]. In future, allogenic transplant will be seriously challenged by less toxic chimeric T-cell approaches described below.

## Checkpoint Blockade Inhibiting Antibodies

Like in other lymphomas, PD-1-blocking antibodies have been in FL tried with varying success. To summarize the attempts from the data available at this time, we start with the curious story of pidilizumab. Pidilizumab was developed by CureTech and was later acquired by Pfizer. For the longest time, it was thought that pidilizumab is a PD-1 targeting antibody and the initial clinical trials showed efficacy and tolerablity compatible with a typical PD-1 antibody profile. In a phase II trial, pidilizumab revealed promising activity in FL. From 29 enrolled patients, 19 had an objective response with a CR in 15/29 (52%) and a partial response (PR) in 4/29 patients (14%) (28).

However, when it came to FDA filing for approval, it was found that the binding of pidilizumab was unclear, and the company had to invest further research to clarify the target. Meanwhile, DLL1 has been identified as the genuine target, and it remains to be seen how this anti-DLL1 antibody will integrate into the treatment landscape of FL.

In a pivotal basket trial of relapsed/refractory non-Hodgkin lymphomas, patients were treated with single-agent nivolumab (29). Here, FL showed the highest objective response rates (40%) followed by DLBCL (36%). Interestingly, in the translational part of the study, the malignant FL cells were mostly negative for PD-L1 and PD-L2. These two antigens, considerably the therapeutic targets, were often expressed on bystander cells in the microenvironment. This fact demands further basic studies to elucidate the mechanism behind checkpoint inhibition in FL.

Regarding checkpoint blockade, it can be expected that a combinational approach of passive immunotherapy (anti-CD20) with anti-PD1 antibodies has potential. At the ASH meeting 2017, first data from a trial combining rituximab with pembrolizumab were shown (30). Here, 30 patients with relapsed FL received rituximab plus pembrolizumab for a total of 16 infusions. With a median observation time of 14 months, no death was noted. The ORR was 67% with a CR rate of 50%. This compares favorably to historical response rates of rituximab of 40%. Currently, a single arm study (NCT03245021) is recruiting and will explore the role of nivolumab in combination with rituximab in first-line therapy of FL. It has to be mentioned the true value of nivolumab may not be determined without a comparator arm of rituximab monotherapy. In the near future, a variety of immunotherapy combination trials will be completed, and it is very likely that the inclusion of checkpoint blockade into standard therapy of FL will improve the outcome of affected patients (for an overview of ongoing trials evaluating novel therapies from 3 to 6, see Table 1).

## Chimeric Antigen Receptor Therapy (CART)

Data regarding CAR therapy in patients with FL is sparse. Especially, early disease and low-grade FL have not been addressed by clinical trials yet. The CAR T cell products relevant to FL treatment are CD19 re-targeted T-cells. These products include axicabtagene ciloleucel/Yescarta ^®^ and Tisagenleleucel/Kymriah^®^ that are FDA approved. The best available information for CAR therapy in FL we have at this moment is from patients participating in the Juliet trial. At ASH 2015, Schuster presented the outcome of 14 FL patients with an ORR of 73% at 3 months with 4 CRs, 4 PRs, and 3 progressive disease. Three of the four PR patients converted into CRs by 6 months and the last patient with PR remained in PR for a year before progression of the disease (31). In an updated analysis encompassing 24 patients, an ORR of 53% was published. At a median follow-up of 28.6 months, sustained remissions were observed and 89% of patients with FL who had an initial response (95% CI, 43–98) could maintain the response (20).

The most notable side effects of CART therapies are cytokine release syndromes found across trials in 50–60%, up to 10% severe (grade 4) and neurological toxicities that appear in frequencies from 25 to 30% and are severe (≥grade 4) in approximately 5%. Neurotoxicity seemed to be associated with the CAR construct itself, as JCAR015 showed higher toxicities than other constructs. In a *post hoc* analysis of the Rocket 1, trial factors associated with higher neurotoxicity were the conditioning chemotherapy (Flu/CY or not) with a higher risk odds ratio of 7.23, the bridging chemotherapy (OR 4,68), age below 30 (OR 5.16), and less or equal 2 previous line of therapies (OR 7.24) (21). No association with higher risk was found regarding prior CNS irradiation, prior IT chemotherapy, prior CNS disease, prior allogeneic transplantation, higher ECOG performance status, or prior use of blinatumumab (21).

With the approval of two CART products in relapsed/refractory aggressive B-cell lymphomas and some 13 trials ongoing (Table 2), the value of the CART approach in FL should become clearer in the next couple of years. If long-lasting remissions can be achieved, this approach has the potential to displace autologous and allogenic stem cell transplantation in FL.

**Table 2:** Ongoing trials chimeric antigen receptor therapy (CART) trials including follicular lymphoma (FL) on clinical.trials.gov.

	Title	Intervention	NCT no.
1	FDG-PET/CT imaging as early predictor of DP	Biological: CART-19 autologous T-cells|radiation: FDG-PET/CT	NCT02476734

2	Treatment of relapsed and/or chemotherapy refractory B-cell malignancy by tandem CAR T cells targeting CD19 and CD22	Biological: anti-CD19/22-CAR vector-transduced T cells	NCT03185494

3	CAR T cell receptor immunotherapy for patients with B-cell lymphoma	Drug: fludarabine|drug: cyclophosphamide|biological: anti-CD19-CAR PBL	NCT00924326

4	Anti-CD22 CAR-T therapy for CD19-refractory or resistant lymphoma patients	Drug: retroviral vector-transduced autologous T cells to express CD22-specific CARs	NCT02721407

5	Memory-enriched CAR-T cells immunotherapy for B cell lymphoma	Drug: CD19.CAR-T cells	NCT02652910

6	Long-term follow-up study for patients previously treated with a juno CAR T-cell product	Genetic: JCAR017|genetic: JCARH125	NCT03436771

7	Competitive transfer of Î ± CD19-TCRz-CD28 and Î ± CD19-TCRz-CD137 CAR-T Cells for B-cell leukemia/lymphoma	Biological: anti-CD19 CAR-T|drug: fludarabine|drug: cyclophosphamide	NCT02685670

8	CAR-T cell immunotherapy in CD19 positive relapsed or refractory leukemia and lymphoma	Biological: PCAR-019 (anti-CD19 CAR-T cells)	NCT02819583

9	CART19 to treat B-cell leukemia or lymphoma that are resistant or refractory to chemotherapy	Biological: CART-19	NCT01029366

10	Treatment of relapsed and/or chemotherapy refractory B-cell malignancy by CART19	Biological: anti-CD19-CAR vector-transduced T cells	NCT01864889

11	Treatment of relapsed and/or chemotherapy refractory B-cell malignancy by tandem CAR T cells targeting CD19 and CD20	Biological: anti-CD19/20-CAR vector-transduced T cells	NCT03097770

12	A safety and efficacy trial of JCAR017 combinations in subjects with relapsed/refractory B-cell malignancies (PLATFORM)	Biological: JCAR017|drug: durvalumab	NCT03310619

13	Study evaluating the safety and pharmacokinetics of JCAR017 in B-cell non-Hodgkin lymphoma (TRANSCEND-NHL-001)	Biological: JCAR017 (lisocabtagene maraleucel) single-dose schedule|biological: JCAR017	NCT02631044

*An overview of the contemporary CART trials that include FL patients, with the experimental intervention and the trial accession number in the second and third row*.

## Summary and Outlook

Follicular lymphoma represents in the most instances an indolent disease and tolerance of the malignant clone by the immune system is very likely. The mutational load that predicts for immune responses appears not to be exceedingly high in this disease. It is quite likely that immunotherapy with checkpoint blockade inhibitors may not find a place in the early course of the disease. However, more aggressive variants, e.g., grade IIIA and higher of FL may represent better targets and should be explored in this regard. Furthermore, during the often long course of the disease, it is reasonable to assume that the malignant clone acquires additional genomic alterations that could make it more prone to respond to checkpoint blockade inhibitors. Finally, FL that transforms into higher grade B-cell lymphoma has a poorer prognosis than *de novo* high-grade B-cell lymphomas. There, a space for immunotherapy on its own or as an adjunct to a standard therapy could be envisioned. However, with a good variety of therapeutic options at hand the role of immunotherapy in the landscape of treating FL has still to be established.

## Author Contributions

CR and FS contributed equally.

## Conflict of Interest Statement

The authors have had roles in advisory boards of Roche (FS and CR), BMS (FS), Celgene (CR), and Janssen (FS and CR).

## Abbreviations

CR, complete response; PR, partial response; PD, progressive disease; FL, follicular lymphoma; OS, overall survival; CART, chimeric antigen receptor therapy.
